# Supplementary material for: Conserved protein Pir2ARS2 mediates gene repression through cryptic introns in lncRNAs
Source: Nat Commun. 2020 May 15;11:2412. doi: 10.1038/s41467-020-16280-y (PMC7229227; doi:10.1038/s41467-020-16280-y)
Supplement: Supplementary file 1 — Supplementary Information [file 41467_2020_16280_MOESM1_ESM.pdf]

Supplementary Information

Conserved protein Pir2^{ARS2} mediates gene repression through cryptic introns in lncRNAs

Thillainadesan et al

Supplementary Figures: 1 – 7

Supplementary Tables: 1 and 2

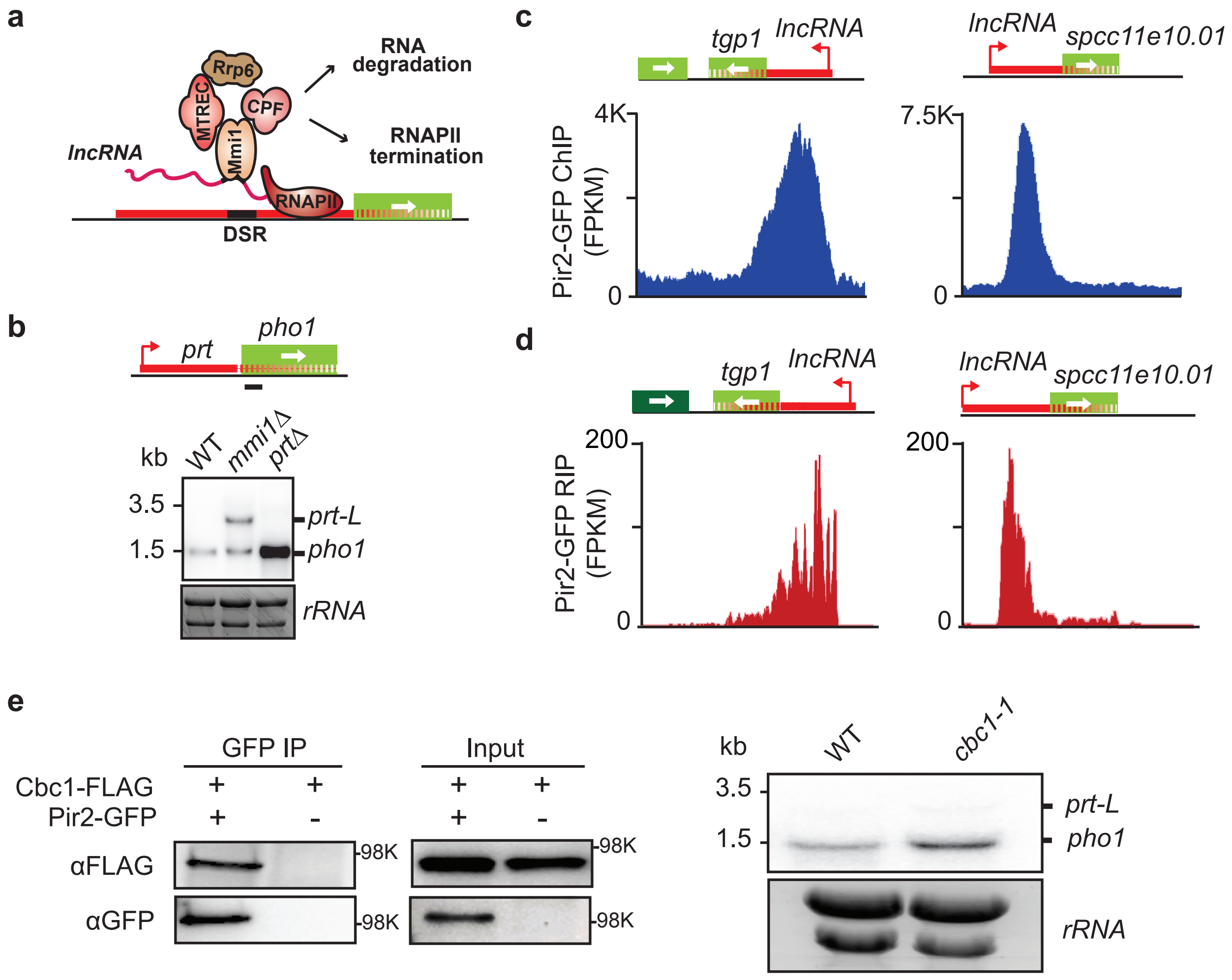

Supplementary Fig. 1. Pir2 and its associated factors target lncRNAs. (a) Diagrammatic representation of RNA processing by the RNA binding protein Mmi1 and its associated factors, such as MTREC, Rrp6 exosome and cleavage polyadenylation factor (CPF), which control termination and degradation of lncRNAs that regulate downstream protein coding genes. **(b)** Northern blot analysis of the *pho1* locus in WT, *mmi1*Δ and *prt*Δ. The radioactive probe is described in Fig. 1a. *prt-L* refers to longer lncRNA transcript. Note that deletion of *prt* causes derepression of the *pho1* gene. Cells were grown in EMM media with phosphate. See Source Data for more details. **(c)** ChIP-seq of Pir2-GFP at *tgp1* and *spcc11e10.01* loci. **(d)** RIP-seq of Pir2-GFP. Schematics of the loci are shown above. **(e)** CBC interacts with Pir2 and is required for lncRNA-mediated gene repression. Co-IP analysis using a strain co-expressing Pir2-GFP and Cbc1-FLAG (Cbc1, a subunit of CBC) proteins (left). Northern blot analysis of the *pho1* locus in WT and *cbc1-1* (right). The radioactive probe used in northern blot analysis is described in Fig 1a.

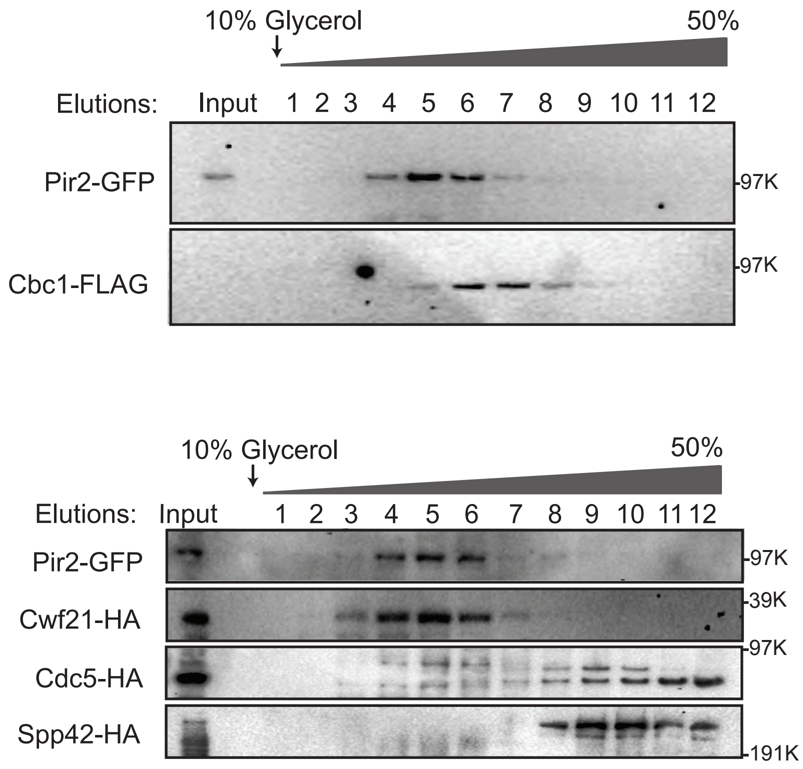

Supplementary Fig. 2. Pir2 co-elutes with CBC and a subset of splicing factors. Glycerol gradient analyses of lysates prepared from strains expressing epitope tagged Pir2, Cbc1 and various splicing factors (Cwf21, Cdc5 and Spp42). Elution fractions are indicated above the gel.

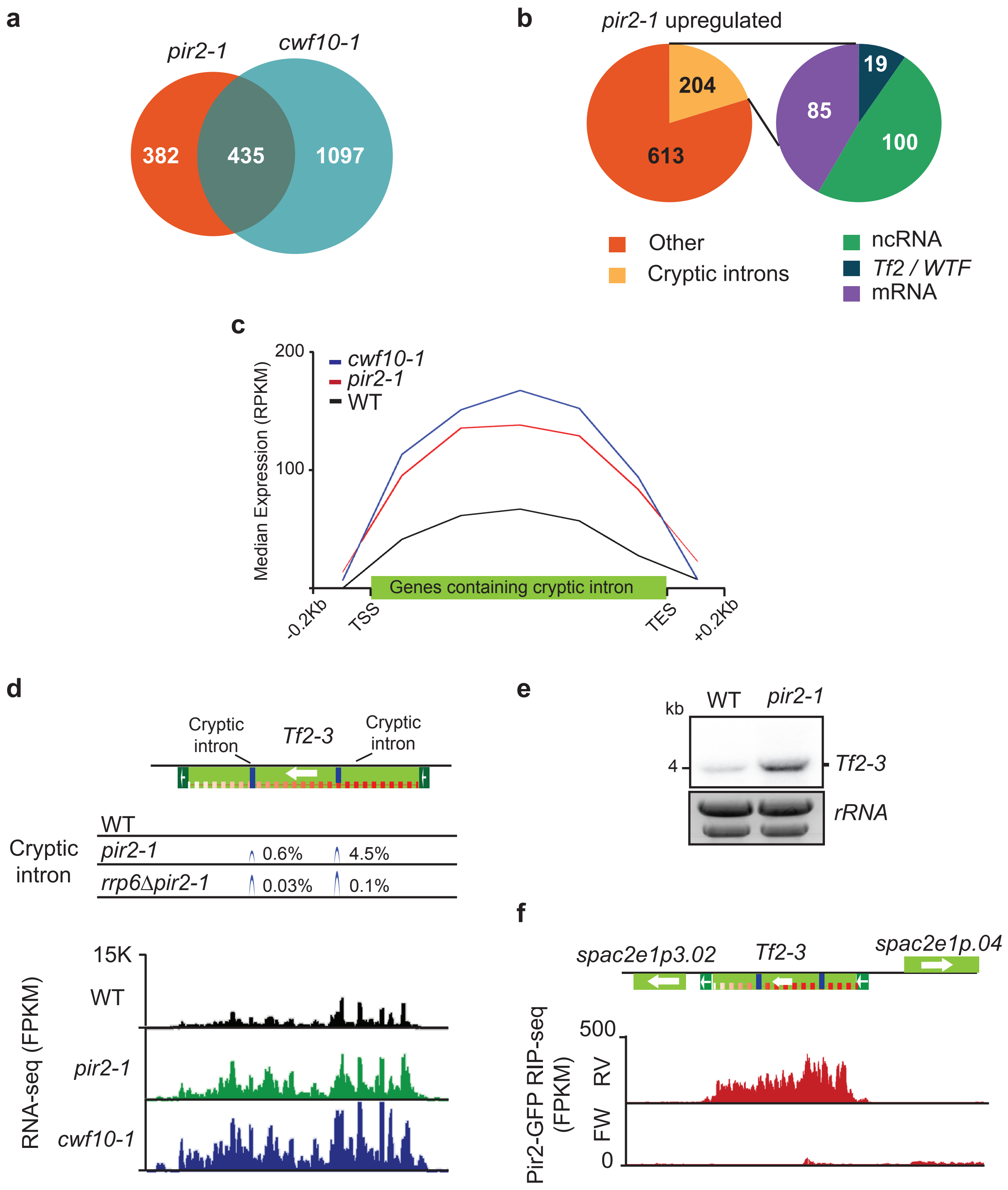

Supplementary Fig. 3. Pir2 and Cwf10 repress a common set of loci genome-wide. (a) Venn diagram of RNA-seq data showing overlap of upregulated genes in *pir2-1* and *cwf10-1* mutants. **(b)** TopHat splice junction analysis of new introns emerging in genes that are upregulated in *pir2-1* (left). Classification of transcripts that contain new introns in *pir2-1* (right). **(c)** RNA-seq median profiles of genes upregulated in *pir2-1* mutant containing cryptic intron(s). **(d)** Normalized (FPKM) RNA-seq signal plotted at the *Tf2-3* locus in WT, *pir2-1* and *cwf10-1*. The panel above depicts the corresponding locus with the cryptic intron marked by a blue box. **(e)** Northern blot analysis of *Tf2-3* in WT and *pir2-1*. **(f)** Normalized (FPKM) Pir2-GFP RIP-seq signal plotted at the *Tf2-3* locus in WT. The RIP-seq signal obtained from the untagged strain was subtracted.

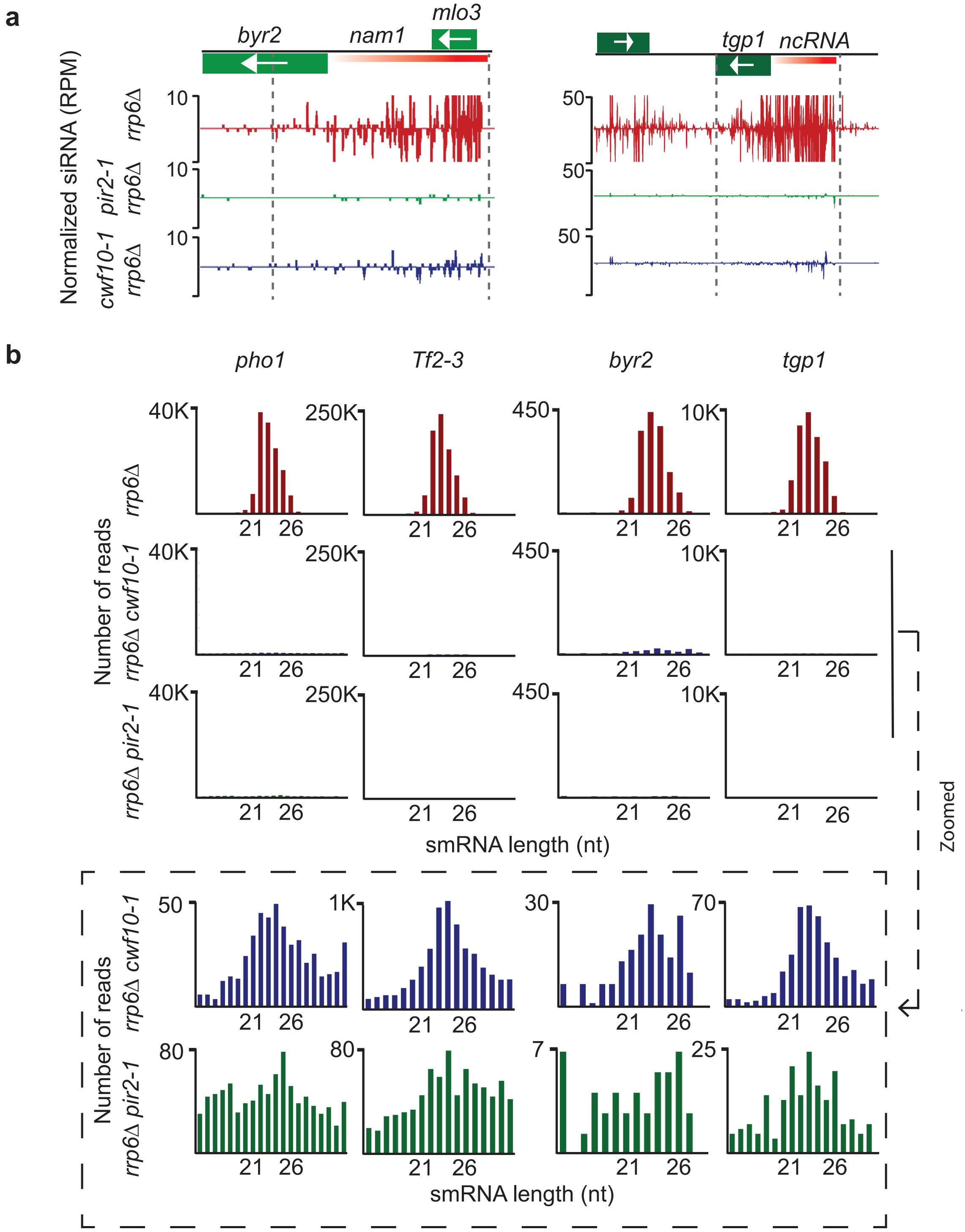

Supplementary Fig. 4. Pir2 and Cwf10 are required for production of siRNA mapping to lncRNAs. (a) Normalized (per million) small RNA-seq signal plotted at the *byr2* and *tgp1* loci. Strains are indicated on the left. **(b)** Read size profiles calculated from small RNA-seq data for the *pho1*, *byr2*, *tgp1*, and *Tf2-3* loci in the indicated strains. The 21 nt and 26 nt bars are indicated under the graph. Zoomed in panels below are included to show read length distribution of small RNAs mapping to indicated loci in mutant cells.

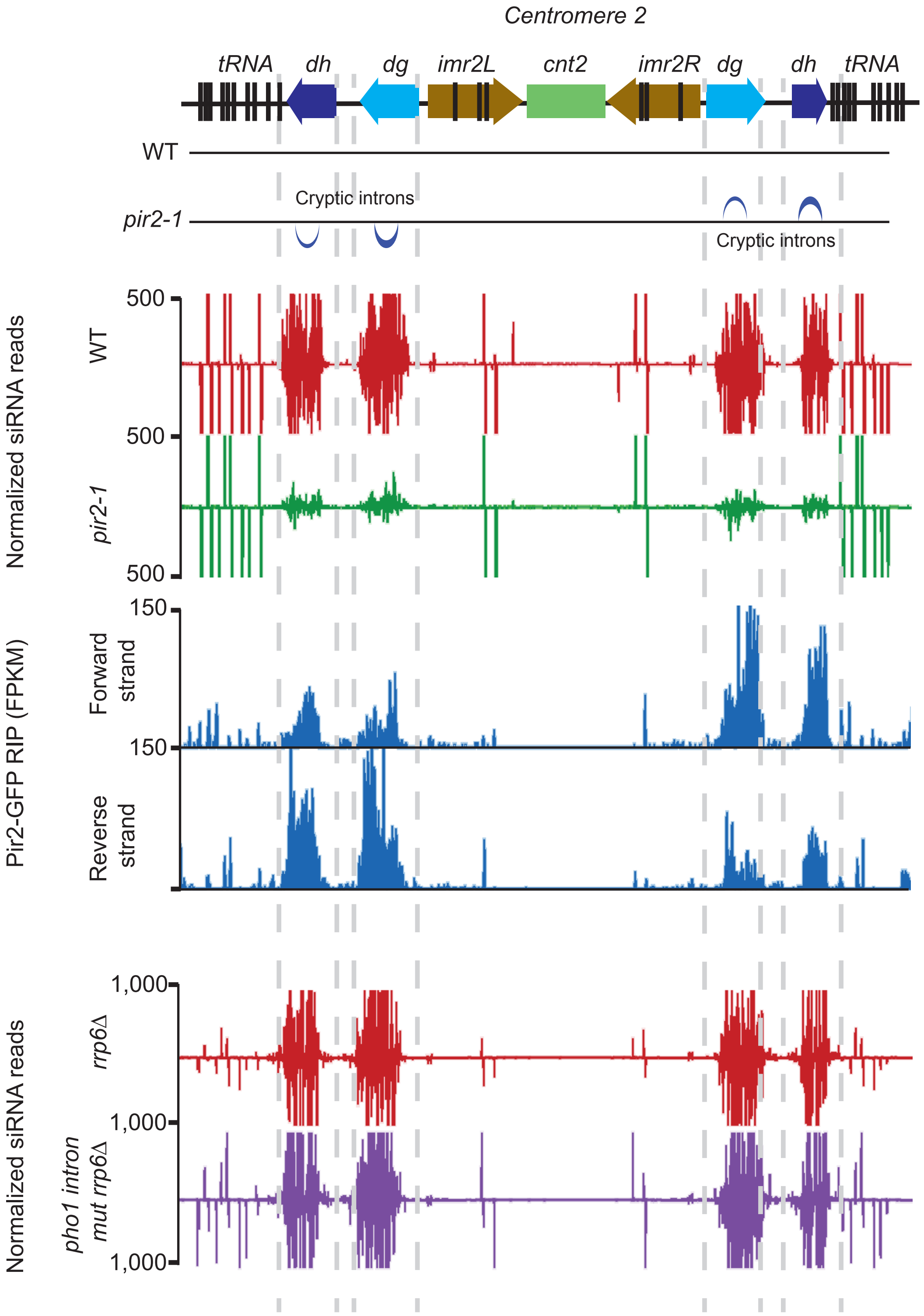

Supplementary Fig. 5. Pir2 binds to centromeric repeats and is required for siRNA production. The cyptic introns, siRNA clusters and Pir2 distribution are shown in alignment with the map of centromere 2 (*cen2*). The central centromere core domain is surrounded by inner and outer pericentromeric repeats. TopHat splice junctions showing the location of introns (blue arcs), siRNA clusters (normalized per million reads) in WT and *pir2-1* strains, and results of RIP-seq analysis of Pir2-GFP are plotted. The levels of siRNAs mapping to pericentromeric repeats are not affected in a strain containing a mutation in the splice sites of a cryptic intron in the *prt-pho1* transcript.

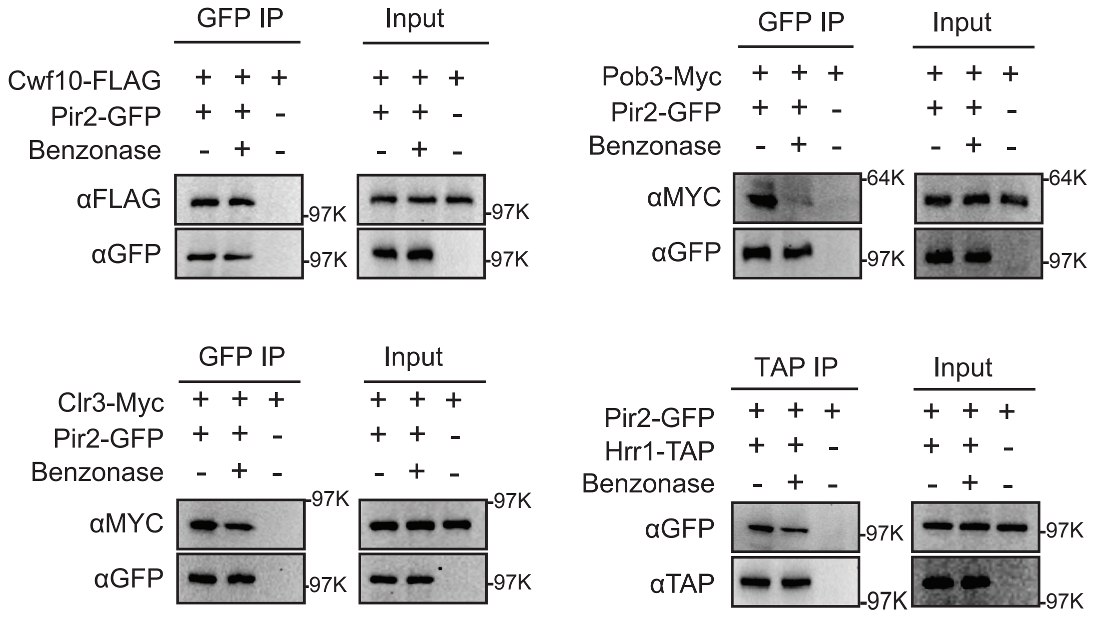

Supplementary Fig. 6. Pir2 co-immunoprecipitation with Benzonase treatment. Co-IP analysis of Pir2-GFP with Cwf10-Flag, Pob3-Myc, Clr3-Myc, and Hrr1-Tap proteins. Lysates treated with Benzonase are indicated. Antibody used for western blotting is indicated to the left of the gel. Antibody used for IP is shown at the top of each gel.

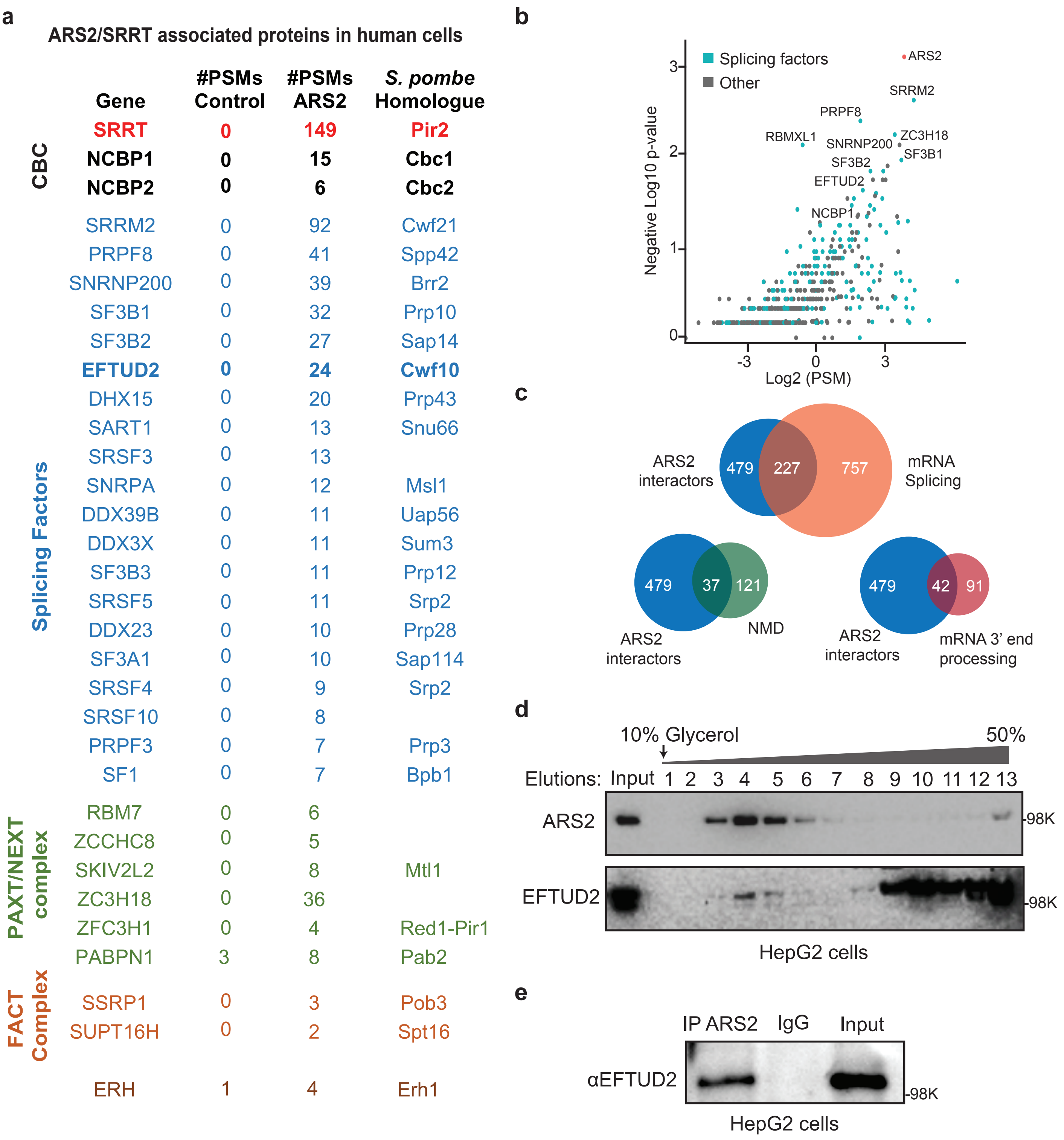

Supplementary Fig. 7. Human ARS2 interacts with splicing factors. (a) RIME (Rapid Immunoprecipitation Mass spectrometry of Endogenous proteins) analysis of ARS2 from HepG2 nuclear extracts. PSM; peptide-spectrum match. **(b)** Volcano plot displaying ARS2 RIME results. P-values for peptide detection were estimated from the empirical distribution of log2((PSMs_ARS2$_N$+1)/(PSMs_IgG$_N$+1)) for all peptides, N, where PSM_ARS2$_N$ is the count of peptide spectrum matches (PSMs) to the Nth peptide in the ARS2 pulldown and PSMs_IgG$_X$ is the corresponding count in the IgG pulldown. The log2(PSMs_ARS2$_N$), normalized for the length of the Nth peptide, is plotted on the X-axis. **(c)** Reactome analysis of the proteins identified in ARS2 RIME. Blue circle represents proteins isolated in the ARS2 purification, orange, mRNA splicing factors; green, Nonsense mediated decay (NMD) factors; and red, 3' end processing factors. Hypergeometric distribution assessment for all three categories yielded a p-value less than 0.0005. **(d)** Glycerol gradient of nuclear extract prepared from HepG2 cells. Fractions containing ARS2 and EFTUD2 were determined using antibodies recognizing the endogenous proteins. **(e)** Co-IP analysis of the interaction between ARS2 and the spliceosome factor EFTUD2 in HepG2 cells.

Supplementary Table 1: Strains used in this study

SPDF842	mat1Msmt0 his2 leu1-32 ade6-210 ura4-d18 mei4ΔC<natMX6 mmi1Δ::hphMX6
SPEN297	mat1Msmt0 his2 leu1-32 ade6-210 ura4-DS/E otr1::ura4^{+} rrp6Δ::kanMX6
SPT305	mat1Msmt0 his2 leu1-32 ade6-210 ura4-DS/E otr1::ura4^{+} red1Δ::kanMX6
SPNL237	mat1Msmt0 his2 leu1-32 ade6-210 ura4-DS/E otr1::ura4^{+} pir2-gfp::kanMX6
SPR156	mat1Msmt0 his2 leu1-32 ade6-210 ura4-DS/E otr1::ura4^{+}
SPDJ93	mat1Msmt0 his2 leu1-32 ade6-210 ura4-DS/E otr1::ura4^{+} pir2-1-flag::kanMX6
SPGT12a	mat1Msmt0 his2 leu1-32 ade6-210 ura4 pir2-gfp::kanMX6 prtΔ::ura4^{+}
SPNL202A	mat1Msmt0 his2 leu1-32 ade6-210 ura4 prtΔ::ura4^{+}
SPF1193	mat1Msmt0 his2 leu1-32 ade6-210 ura4-DS/E otr1::ura4^{+} cwf10-flag::kanMX6
SPGT21	mat1Msmt0 his2 leu1-32 ade6-210 ura4-DS/E otr1::ura4^{+} cwf10-flag::kanMX6 pir2-gfp::kanMX6
SPF1152	mat1Msmt0? leu1-32 ade6-210 ura4-d18 cwf10-1 otr1::ura4^{+}
SPGT9	mat1Msmt0 his2 leu1-32 ade6-210 ura4-DS/E rrp6Δ::kanMX6 pir2-1-flag::kanMX6
SPSH1173a	h^{90} leu1-32 ade6-210 ura4 pir2-1-flag::kanMX6
SPSH1174a	h^{90} leu1-32 ade6-216 ura4 rrp6Δ::kanMX6
SPSH1175a	h^{90} leu1-32 ade6-216 ura4 rrp6Δ::kanMX6 pir2-1-flag::kanMX6
SPSH1176a	h^{90} leu1-32 ade6-216 ura4
SPGT134	mat1Msmt0 his2 leu1-32 ade6-210 ura4-DS/E pho1 cryptic intron mutant
SPGT135	mat1Msmt0 his2 leu1-32 ade6-210 ura4-DS/E pho1 cryptic intron mutant
SPGT35	mat1Msmt0 his2 leu1-32 ade6-216 ura4-DS/E pir2-1-flag::kanMX6 cwf10-1
SPGT137	mat1Msmt0 his2 leu1-32 ade6-210 ura4-DS/E rrp6Δ pho1 cryptic intron mutant
SPGT2b	mat1Msmt0 his2 leu1-32 ade6-210 ura4-DS/E otr1::ura4^{+} pir2-gfp::kanMX6 hrr1-TAP::kanMX6
SPGT15	mat1Msmt0 his2 leu1-32 ade6-216 ura4-DS/E otr1::ura4^{+} pob3-myc::kanMX6 pir2-gfp::kanMX6
SPGT42	mat1Msmt0 his2 leu1-32 ade6-216 ura4-DS/E pob3-myc::kanMX6 pir2-1-flag::kanMX6
SPGT74	mat1Msmt0 his2 leu1-32 ade6-216 ura4-DS/E clr3-myc::kanMX6 pir2-1-flag::kanMX6
SPKZ227	mat1Msmt0 his2 leu1-32 ade6-216 ura4-DS/E otr1::ura4^{+} pob3-myc::kanMX6
SPGT25	mat1Msmt0 his2 leu1-32 ade6-216 ura4-DS/E pir2-gfp::kanMX6 clr3-myc::kanMX6
SPT1929	mat1Msmt0 his2 leu1-32 ade6-210 ura4-D18 otr1::ura4^{+} clr3-myc::kanMX6

SPGT48	*mat1Msmt0 his2 leu1-32 ade6-216 ura4-DS/E otr1::ura4$^+$ pir2-1-flag::kanMX6 clr3Δ::kanMX6*
SPVC240	*mat1Msmt0 his2 leu1-32 ade6-210 ura4-DS/E otr1::ura4$^+$ clr3Δ::kanMX6*
SPKZ374a	*mat1Msmt0 his2 leu1-32 ade6-216 ura4-DS/E otr1::ura4$^+$ pob3Δ::kanMX6*
SPJ71	*mat1Msmt0 his2 leu1-32 ade6-210 ura4-DS/E otr1::ura4$^+$ ago1Δ::kanMX6*
SPGT136	*mat1Msmt0 his2 leu1-32 ade6-210 ura4-DS/E otr1::ura4$^+$ pir2-1-flag::kanMX6 pho1 cryptic intron mutant*
SPGT119	*mat1Msmt0 his2 leu1-32 ade6-210 ura4-DS/E pir2-1-flag::kanMX6 ago1Δ::kanMX6*
SPF261A	*mat1Msmt0 his2 leu1-32 ade6-210 ura4-DS/E otr1::ura4$^+$ ago1Δ::kanMX6 clr3Δ::kanMX6*
SPSH1230a	*h^{90} leu1-32 ade6::kanMX6 ura4-DS/E rrp6Δ::kanMX6 cwf10-1 otr1::ura4$^+$*
SPSH1238a	*h^{90} leu1-32 ade6::kanMX6 otr1::ade6$^+$ lys1::natR ura4-D18 cwf10-1*
SPGT146	*mat1Msmt0 his2 leu1-32 ade6-210 ura4-DS/E otr1::ura4$^+$ cwf21-HA::hphMX6 pir2-gfp::kanMX6*
SPGT148	*mat1Msmt0 his2 leu1-32 ade6-210 ura4-DS/E otr1::ura4$^+$ spp42-HA::hphMX6 pir2-gfp::kanMX6*
SPGT150	*mat1Msmt0 his2 leu1-32 ade6-210 ura4-DS/E otr1::ura4$^+$ cdc5-HA::hphMX6 pir2-gfp::kanMX6*
SPGT129	*mat1Msmt0 his2 leu1-32 ade6-216 ura4-DS/E otr1::ura4$^+$ cbc1-1-flag::kanMX6*
SPGT109	*mat1Msmt0 his2 leu1-32 ade6-210 ura4-DS/E otr1::ura4$^+$ cbc1-flag::kanMX6 pir2-gfp::kanMX6*
SPGT120	*mat1Msmt0 his2 leu1-32 ade6-210 ura4-DS/E otr1::ura4$^+$ cbc1-flag::kanMX6*
SPGT126	*mat1Msmt0 his2 leu1-32 ade6-210 ura4-DS/E otr1::ura4$^+$ hrr1-TAP::kanMX6 pir2-gfp::kanMX6 cwf10-1*

Supplementary Table 2: Primers used in this study

**Northern probe synthesis**	
pho1Fwd1	ATTCCTTGGCTTTTTGGCCG
pho1T7Rev2	TAATACGACTCACTATAGGGCTGGGCGGCAGTGTAAATGT
*byr2*_nor_F	CCCAATTTCTCCAACTTCCA
*byr2*_nor_R	TAATACGACTCACTATAGGATTGTAAGCAATCCGGCAAC
tf2-1 fwd1	GAGCAAAACGATGGAGACCAGAGA
tf2-1 T7-rev1	TAATACGACTCACTATAGGGCACTGATTTCTGATTTCCAGTAATTTCTTTCC
**RT-qPCR, ChIP-qPCR primers and Strain construction oligos**	
*pho1*_QPCR_F	ACGCTCAGTTCGCTGAATTT
*pho1*_QPCR_R	GTGGTGGGAAAGTCAATGCT
*byr2*_QPCR_F	AATTCATCTTCACCGGAACG
*byr2*_QPCR_R	GCCAATTCAATCTCCCAAGA
pho1-2-fwd (prt)	GATTTGCTGTTTGGAAAATTAGG
pho1-2-rev (prt)	CCTGTTGTTCAAACATGTCC
tf2-3-s	TCAACCAACAAATGACTTGA
tf2-3-as	CGAGCCTAGATTCAAAGAAA
qPCR-*leu1*-fwd	CCTAAGGAGGCTGAAGCTATCG
qPCR-*leu1*-rev	TCGCGAGTATAAAGACCACGTC
*cwf21*_pfatag_F	GCAATTCACCCTCCTATACGAAGAATGAGTCAATTCCTGTTGTGGACAGGGATTCCTCTCCTGAGGGTGGCGAAATTGTACGGATCCCCGGGTTAATTAA
*cwf21*_pfatag_R	TATCCAATATTAATAAACAATACATGCTTGCATGAATTGCCTATTATATTATTGTTTTAACTGGCCTAACTCTTTGGGCCGAATTCGAGCTCGTTTAAAC
*spp42*_pfatag_F	ATAGACCTACTCATGTAATAAGCTATACTGAACTGGAGACGAATGACAGATTAGAGGAAGATATGCCAGATGCATTTGCACGGATCCCCGGGTTAATTAA
*spp42*_pfatag_R	CATAAAGCACTCAAATTAGCAAAAATCAAAATATACATTAATTGCCCAAAATCCAGTTACGAAAATAAATAGCTCCTCATGAATTCGAGCTCGTTTAAAC
*cdc5*_pfatag_F	CAGCTGAAATAGAGCTAAGCAAAATGCAACAAATCGAGGCGTATGCTCAACAAGATTATGCTAGGGTTACTGGACAAAATCGGATCCCCGGGTTAATTAA
*cdc5*_pfatag_R	TTTCGTTTTATTTCAGGCATGATAATATACACTATGAATGATAAGCTATGACAATTATTTAAAACAAAGTGAAGTGAAGTGAATTCGAGCTCGTTTAAAC
cbc1-F-PFA6	AAATGCAACCCGTCGACGCAGTAGATGAGCAGCCATCAGAAAATAATCAAACAGCAGCTGATGCAACTAATGAGGAAAAACGGATCCCCGGGTTAATTAA
cbc1-R-PFA6	GATTACCAGTCGAGTTCTTCAGTTTAATCTTTTTGATATCAAAACAACACTTTCTTCATTTTTGTTATAGACTACTTTCTGAATTCGAGCTCGTTTAAAC
