# Supplementary material for: Conserved protein Pir2ARS2 mediates gene repression through cryptic introns in lncRNAs
Source: Nat Commun. 2020 May 15;11:2412. doi: 10.1038/s41467-020-16280-y (PMC7229227; doi:10.1038/s41467-020-16280-y)
Supplement: Supplementary file 3 — Reporting Summary [file 41467_2020_16280_MOESM3_ESM.pdf]

nature research

Corresponding author(s): Shiv I. S. Grewal
Last updated by author(s): Apr 8, 2020

Reporting Summary

Nature Research wishes to improve the reproducibility of the work that we publish. This form provides structure for consistency and transparency in reporting. For further information on Nature Research policies, see Authors & Referees and the Editorial Policy Checklist.

Statistics

For all statistical analyses, confirm that the following items are present in the figure legend, table legend, main text, or Methods section.

n/a	Confirmed	
☐	☒	The exact sample size (*n*) for each experimental group/condition, given as a discrete number and unit of measurement
☐	☒	A statement on whether measurements were taken from distinct samples or whether the same sample was measured repeatedly
☐	☒	The statistical test(s) used AND whether they are one- or two-sided<br>*Only common tests should be described solely by name; describe more complex techniques in the Methods section.*
☒	☐	A description of all covariates tested
☒	☐	A description of any assumptions or corrections, such as tests of normality and adjustment for multiple comparisons
☐	☒	A full description of the statistical parameters including central tendency (e.g. means) or other basic estimates (e.g. regression coefficient) AND variation (e.g. standard deviation) or associated estimates of uncertainty (e.g. confidence intervals)
☐	☒	For null hypothesis testing, the test statistic (e.g. *F*, *t*, *r*) with confidence intervals, effect sizes, degrees of freedom and *P* value noted<br>*Give P values as exact values whenever suitable.*
☒	☐	For Bayesian analysis, information on the choice of priors and Markov chain Monte Carlo settings
☒	☐	For hierarchical and complex designs, identification of the appropriate level for tests and full reporting of outcomes
☒	☐	Estimates of effect sizes (e.g. Cohen's *d*, Pearson's *r*), indicating how they were calculated

Our web collection on statistics for biologists contains articles on many of the points above.

Software and code

Policy information about availability of computer code

Data collection	MiSeq v3.1 software (illumina) was used to collect RNAseq and smRNAseq data from MiSEQ (illumina) sequencer.<br>NextSeq System Suite v2.2.0 was used to collect RIPseq and ChIPseq data.<br>Typhoon FLA 9500 control software was used to collect northern blot images.<br>DeltaVision Elite fluorescence microscope (Applied Precision, GE Healthcare) with Olympus 100x/1.40 objective.
Data analysis	R (3.6.1) was used for RNAseq analysis.<br>deepTools (3.1.3) was used to normalize RNAseq/RIPseq/ChIPseq data and generate bedgraphs.<br>MACs was used to identify and call peaks for ChIPseq.<br>TopHAT (2.1.1) was used for RNAseq and RIPseq alignment.<br>Novoalign (V2) was used for smRNA alignment.<br>Bowtie2(2.3.4) was used for ChIPseq alignment.<br>SequestHT v1.17 and Proteome Discoverer 2.2 (Thermo) used to search MS the data against human proteins.<br>SoftWoRx v7.0 was used to capture images from DeltaVision.<br>Fiji ImageJ V.1.0 was used to analyze microscopy images

For manuscripts utilizing custom algorithms or software that are central to the research but not yet described in published literature, software must be made available to editors/reviewers. We strongly encourage code deposition in a community repository (e.g. GitHub). See the Nature Research guidelines for submitting code & software for further information.

Data

Policy information about availability of data

All manuscripts must include a data availability statement. This statement should provide the following information, where applicable:

- Accession codes, unique identifiers, or web links for publicly available datasets
- A list of figures that have associated raw data
- A description of any restrictions on data availability

All authors declare that the data supporting the findings in this work are available within the maintext, figures and supplementary figures. Raw datasets are available on NCBI Gene Expression Omnibus (Accession no. GSE135161) for RNA and DNA sequencing data. ASM294v2 genome was used in this study to align RNA and DNA sequencing from www.pombase.org. The mass spectrometry proteomics data for RIME have been deposited to the ProteomeXchange Consortium via the PRIDE partner repository with the dataset identifier PXD018373 and 10.6019/PXD018373. The source data underlying Figs 1a, b, e, 2a-d, 3c, d, 4a, b, e, and 5a-e and Supplementary Figs 1b, e, 2, 3e, 6, 7d, e are provided as a Source Data file.

Field-specific reporting

Please select the one below that is the best fit for your research. If you are not sure, read the appropriate sections before making your selection.

☒ Life sciences ☐ Behavioural & social sciences ☐ Ecological, evolutionary & environmental sciences

For a reference copy of the document with all sections, see nature.com/documents/nr-reporting-summary-flat.pdf

Life sciences study design

All studies must disclose on these points even when the disclosure is negative.

Sample size	Three biological replicates were chosen as the n number for each qPCR experiment to ensure calculation of statistical significance.
Data exclusions	No data was excluded.
Replication	Experiments were repeated at least two times and the results successfully yielded similar or identical result.
Randomization	Randomly selected samples were allocated into experimental groups.
Blinding	Experimental biases were not expected based on the type of experiments conducted. So therefore, no blinding was necessary for this study.

Reporting for specific materials, systems and methods

We require information from authors about some types of materials, experimental systems and methods used in many studies. Here, indicate whether each material, system or method listed is relevant to your study. If you are not sure if a list item applies to your research, read the appropriate section before selecting a response.

Materials & experimental systems

n/a	Involved in the study
☐	☒ Antibodies
☐	☒ Eukaryotic cell lines
☐	☐ Palaeontology
☐	☐ Animals and other organisms
☐	☐ Human research participants
☐	☐ Clinical data

Methods

n/a	Involved in the study
☐	☒ ChIP-seq
☐	☐ Flow cytometry
☐	☐ MRI-based neuroimaging

Antibodies

Antibodies used	anti-FLAG (M2 Sigma, F1804), anti-GFP (Roche, 11814460001), anti-c-MYC (Covance, 9E10), anti-cMYC gel (Sigma, A7470), anti-EFTUD2 (Abcam, ab72456), anti-ARS2 antibody (Abcam, ab192999), anti-GFP (Abcam, ab290), anti-HA (Biolegend, 901501), Rabbit IgG (Abcam, ab37415).
Validation	According to manufacturer's technical antibody sheet, the antibodies were validated for their appropriate use.<br>anti-GFP and ant-MYC validated for ChIP and all other antibodies were validated for IP and western blotting.

Eukaryotic cell lines

Policy information about cell lines

Cell line source(s)	American Type Culture Collection (ATCC). HepG2 ATCC (ATCC HB-8065).
Authentication	Was validated by the provider (ATCC).
Mycoplasma contamination	Mycoplasma tests were done using Hoechst and direct culture method followed by microscopy techniques and the results were confirmed to be negative for Mycoplasma.
Commonly misidentified lines (See ICLAC register)	No commonly mis-identified strain was used in this study.

Palaeontology

Specimen provenance	n/a
Specimen deposition	n/a
Dating methods	n/a

☐ Tick this box to confirm that the raw and calibrated dates are available in the paper or in Supplementary Information.

Animals and other organisms

Policy information about studies involving animals; ARRIVE guidelines recommended for reporting animal research

Laboratory animals	n/a
Wild animals	n/a
Field-collected samples	n/a
Ethics oversight	n/a

Note that full information on the approval of the study protocol must also be provided in the manuscript.

Human research participants

Policy information about studies involving human research participants

Population characteristics	n/a
Recruitment	n/a
Ethics oversight	n/a

Note that full information on the approval of the study protocol must also be provided in the manuscript.

Clinical data

Policy information about clinical studies

All manuscripts should comply with the ICMJE guidelines for publication of clinical research and a completed CONSORT checklist must be included with all submissions.

Clinical trial registration	n/a
Study protocol	n/a
Data collection	n/a
Outcomes	n/a

ChIP-seq

Data deposition

☒ Confirm that both raw and final processed data have been deposited in a public database such as GEO.

☒ Confirm that you have deposited or provided access to graph files (e.g. BED files) for the called peaks.

Data access links<br>*May remain private before publication.*	The following GEO token can be used to access the data: mfkxymegvfcrjgn for GSE: GSE135161
Files in database submission	Fastq and bedgraph
Genome browser session<br>(e.g. UCSC)	n/a

Methodology

Replicates	2 biological replicates were performed.
Sequencing depth	~20M reads were sequenced and 90 percent which was uniquely aligned. Read length = 75bps single end reads. MACs was used to identify and call peaks.
Antibodies	Anti-GFP (Abcam, ab290)
Peak calling parameters	MACs was used to identify high confidence peaks.
Data quality	An FDR < 1% and fold enrichment >2 was used and only high confidence peaks are relevant for this study.
Software	Bowtie2(2.3.4) was used for ChIPseq alignment and deepTools (3.1.3) was used for generation of bedgraphs.

Flow Cytometry

Plots

Confirm that:

☐ The axis labels state the marker and fluorochrome used (e.g. CD4-FITC).

☐ The axis scales are clearly visible. Include numbers along axes only for bottom left plot of group (a 'group' is an analysis of identical markers).

☐ All plots are contour plots with outliers or pseudocolor plots.

☐ A numerical value for number of cells or percentage (with statistics) is provided.

Methodology

Sample preparation	*Describe the sample preparation, detailing the biological source of the cells and any tissue processing steps used.*
Instrument	*Identify the instrument used for data collection, specifying make and model number.*
Software	*Describe the software used to collect and analyze the flow cytometry data. For custom code that has been deposited into a community repository, provide accession details.*
Cell population abundance	*Describe the abundance of the relevant cell populations within post-sort fractions, providing details on the purity of the samples and how it was determined.*
Gating strategy	*Describe the gating strategy used for all relevant experiments, specifying the preliminary FSC/SSC gates of the starting cell population, indicating where boundaries between "positive" and "negative" staining cell populations are defined.*

☐ Tick this box to confirm that a figure exemplifying the gating strategy is provided in the Supplementary Information.

Magnetic resonance imaging

Experimental design

Design type	*Indicate task or resting state; event-related or block design.*
Design specifications	*Specify the number of blocks, trials or experimental units per session and/or subject, and specify the length of each trial or block (if trials are blocked) and interval between trials.*
Behavioral performance measures	*State number and/or type of variables recorded (e.g. correct button press, response time) and what statistics were used*

Behavioral performance measures	*to establish that the subjects were performing the task as expected (e.g. mean, range, and/or standard deviation across subjects).*

Acquisition

Imaging type(s)	*Specify: functional, structural, diffusion, perfusion.*
Field strength	*Specify in Tesla*
Sequence & imaging parameters	*Specify the pulse sequence type (gradient echo, spin echo, etc.), imaging type (EPI, spiral, etc.), field of view, matrix size, slice thickness, orientation and TE/TR/flip angle.*
Area of acquisition	*State whether a whole brain scan was used OR define the area of acquisition, describing how the region was determined.*

Diffusion MRI ☐ Used ☐ Not used

Preprocessing

Preprocessing software	*Provide detail on software version and revision number and on specific parameters (model/functions, brain extraction, segmentation, smoothing kernel size, etc.).*
Normalization	*If data were normalized/standardized, describe the approach(es): specify linear or non-linear and define image types used for transformation OR indicate that data were not normalized and explain rationale for lack of normalization.*
Normalization template	*Describe the template used for normalization/transformation, specifying subject space or group standardized space (e.g. original Talairach, MNI305, ICBM152) OR indicate that the data were not normalized.*
Noise and artifact removal	*Describe your procedure(s) for artifact and structured noise removal, specifying motion parameters, tissue signals and physiological signals (heart rate, respiration).*
Volume censoring	*Define your software and/or method and criteria for volume censoring, and state the extent of such censoring.*

Statistical modeling & inference

Model type and settings	*Specify type (mass univariate, multivariate, RSA, predictive, etc.) and describe essential details of the model at the first and second levels (e.g. fixed, random or mixed effects; drift or auto-correlation).*
Effect(s) tested	*Define precise effect in terms of the task or stimulus conditions instead of psychological concepts and indicate whether ANOVA or factorial designs were used.*

Specify type of analysis: ☐ Whole brain ☐ ROI-based ☐ Both

Statistic type for inference (See Eklund et al. 2016)	*Specify voxel-wise or cluster-wise and report all relevant parameters for cluster-wise methods.*
Correction	*Describe the type of correction and how it is obtained for multiple comparisons (e.g. FWE, FDR, permutation or Monte Carlo).*

Models & analysis

n/a	Involved in the study
☐	☐ Functional and/or effective connectivity
☐	☐ Graph analysis
☐	☐ Multivariate modeling or predictive analysis

Functional and/or effective connectivity	*Report the measures of dependence used and the model details (e.g. Pearson correlation, partial correlation, mutual information).*
Graph analysis	*Report the dependent variable and connectivity measure, specifying weighted graph or binarized graph, subject- or group-level, and the global and/or node summaries used (e.g. clustering coefficient, efficiency, etc.).*
Multivariate modeling and predictive analysis	*Specify independent variables, features extraction and dimension reduction, model, training and evaluation metrics.*
