# Supplementary material for: Conserved protein Pir2ARS2 mediates gene repression through cryptic introns in lncRNAs
Source: Nat Commun. 2020 May 15;11:2412. doi: 10.1038/s41467-020-16280-y (PMC7229227; doi:10.1038/s41467-020-16280-y)
Supplement: Supplementary file 4 — Description of Additional Supplementary Files [file 41467_2020_16280_MOESM4_ESM.pdf]

Description of Additional Supplementary Files

File Name: Supplementary Data 1
Description: RNA-seq analysis of pir2-1 and cwf10-1.

File Name: Supplementary Data 2
Description: List of loci showing small RNA clusters in different genetic backgrounds.

File Name: Supplementary Data 3
Description: Results of RIME analysis of ARS2.
